# The Role of Resistin in Macrovascular and Microvascular Complications of Type 2 Diabetes

**DOI:** 10.3390/life15040585

**Published:** 2025-04-02

**Authors:** Africa Samantha Reynoso-Roa, Susan Andrea Gutiérrez-Rubio, Ezequiel Magallón-Gastélum, Trinidad García-Iglesias, Daniel Osmar Suárez-Rico, Teresa Arcelia García-Cobián

**Affiliations:** 1 Centro Universitario de Ciencias de la Salud, Universidad de Guadalajara, Guadalajara 44340, Mexico; africa.reynoso0835@alumnos.udg.mx; 2 Departamento de Fisiología, Centro Universitario de Ciencias de la Salud, Universidad de Guadalajara, Calle Sierra Mojada 950, Independencia Oriente, Guadalajara 44340, Mexico; susan.gutierrez@academicos.udg.mx (S.A.G.-R.); daniel.suarez@academicos.udg.mx (D.O.S.-R.); 3 Departamento de Salud Pública, Centro Universitario de Ciencias de la Salud, Universidad de Guadalajara, Calle Sierra Mojada 950, Independencia Oriente, Guadalajara 44340, Mexico; ezequiel.magallon@academicos.udg.mx; 4 Instituto de Investigación de Cáncer en la Infancia y Adolescencia (INICIA), Centro Universitario de Ciencias de la Salud, Universidad de Guadalajara, Calle Sierra Mojada 950, Independencia Oriente, Guadalajara 44340, Mexico; trinidad.giglesias@academicos.udg.mx

**Keywords:** resistin, chronic complications, type 2 diabetes

## Abstract

Resistin is an adipokine produced in adipose tissue with pro-inflammatory properties, whose elevation has been associated with insulin resistance and diabetes. Over the past years, significant research has explored the pathophysiological mechanisms involving resistin, utilizing various in vitro and in vivo models. Additionally, numerous clinical studies have aimed to establish a correlation between resistin and the development and progression of macrovascular and microvascular complications in type 2 diabetes. This narrative review summarizes in vitro, in vivo, and human studies published in English since the discovery of resistin in 2001 to the present, examining the role of this adipokine in the pathophysiology of macrovascular and microvascular complications in in vivo and in vitro T2D models, as well as the clinical evidence supporting its use as a biochemical marker in patients with these conditions. The results exhibit considerable heterogeneity and appear to be dependent on the experimental model or population studied. While experimental evidence supports resistin’s involvement at the cellular and molecular levels in the pathogenesis of these complications, current clinical evidence remains insufficient to justify its use as a biochemical marker for either diagnosis or prognosis. Therefore, further well-designed studies are required to elucidate resistin’s potential role in the clinical setting.

## 1. Introduction

In the management of diabetes, healthcare efforts should focus on preventing and addressing chronic complications, as they account for a significant portion of mortality in patients with type 2 diabetes. These complications can be categorized as macrovascular or microvascular, with major conditions including hypertension, atherosclerosis, coronary heart disease (CHD), acute myocardial infarction (AMI), heart failure, diabetic retinopathy, and diabetic nephropathy.

Resistin is an adipokine produced by adipose tissue, belonging to a family of molecules known as FIZZ (*Found in Inflammatory Zone*) proteins. It is a C-terminal domain polypeptide consisting of 108 amino acids, with a molecular weight of 12.5 kDa and a structure rich in cysteine, and it is encoded by the RETN gene located on chromosome 19 [1]. In mice, this protein is primarily expressed by adipose tissue and is readily detectable in serum. Its human counterpart is also expressed in adipocytes, albeit at lower levels. However, the majority of circulating resistin in human plasma is produced by macrophages and monocytes in both blood and adipose tissue in response to inflammatory stimuli, such as increased lipopolysaccharides (LPSs) and other pro-inflammatory cytokines, primarily TNF-α, IL-6, and IL-1β [1].

Toll-like receptor 4 (TLR4) and adenylate-cyclase-associated protein 1 (CAP1) have been identified as the main receptors for resistin, through which it exerts its cellular effects. The pro-inflammatory effects of resistin are induced upon its binding to TLR4 on macrophages, leading to the subsequent activation of the JNK and p38 MAPK pathways. This signaling cascade ultimately results in the translocation of nuclear factor-kB (NF-kB) and the secretion of TNF-α, IL-6, and IL-1β, creating a cytokine-rich pro-inflammatory environment. If this condition becomes chronic, it can lead to deleterious effects on local tissues and contribute to the development of various metabolic disorders [2,3].

In addition to its pro-inflammatory effects, resistin acts as a hormone that antagonizes insulin action. Accumulating evidence over the years suggests that its dysregulation may play a significant role in the pathogenesis of chronic diseases such as type 2 diabetes (T2D) [1,2]. It is well established that inflammation is a major component of the pathogenesis of this disease. In obesity, excess adipose tissue and increased production of adipokines—such as tumor necrosis factor-alpha (TNF-α), various interleukins, and resistin—trigger a state of chronic inflammation [1,2]. Resistin itself directly promotes the increased production of TNF-α and IL-6, creating a pro-inflammatory cytokine-rich environment that leads to mitochondrial damage, oxidative stress, and cellular apoptosis. These processes serve as a precursor to the development of IR and, consequently, T2D [2,3].

Current clinical evidence suggests that resistin levels tend to increase in the presence of metabolic disorders such as obesity, metabolic syndrome, insulin resistance (IR), and type 2 diabetes (T2D). This elevation appears to be correlated with oxidative stress, chronic inflammation, and the insulin resistance that coexist in these conditions. Studies have demonstrated that serum resistin levels are higher in patients with insulin resistance compared to healthy individuals, with an even more pronounced increase in those diagnosed with T2D [4,5].

However, multiple studies in different populations have reported contradictory results when attempting to correlate serum resistin levels with obesity, IR, and T2D. Some studies have identified positive correlations between elevated serum resistin and T2D, while others found no significant association [6]. A meta-analysis published in 2019 concluded that there is a relationship between serum resistin levels and IR in patients with T2D and obesity; however, this conclusion was applicable only to subgroups of individuals with above-average resistin levels. Nevertheless, specific thresholds defining elevated resistin levels in the bloodstream have not been established, as the meta-analysis highlighted significant heterogeneity among the results [7].

Broadly, T2D complications can be classified as macrovascular and microvascular. Among the most significant are hypertension, atherosclerosis, coronary heart disease (CHD), acute myocardial infarction (AMI), heart failure, diabetic retinopathy, and diabetic nephropathy [8,9]. Similar to the rising incidence of T2D, the frequency of its complications is also increasing, representing the primary causes of morbidity and mortality in patients with diabetes [10]. These complications are largely driven by the deterioration in vascular structure and function, induced by hyperglycemia and oxidative stress, both hallmarks of T2D.

The need to prevent and diagnose these diabetes-associated comorbidities early has led to the investigation of new molecular markers that could serve as prognostic factors or therapeutic targets for the treatment of chronic complications of diabetes. Adipose tissue produces molecules that appear to influence the pathogenesis and progression of vascular damage associated with T2D, including resistin, which plays a role in regulating the balance between pro-inflammatory and anti-inflammatory effects. However, in T2D, this balance is disrupted, leading to a shift in the adipokine secretion pattern towards a pro-inflammatory profile. Given that elevated resistin levels have been observed in patients with diabetes, it is proposed that resistin may play a key role in the development of T2D-related complications by contributing to IR, promoting vascular injury, and consequently, vascular disease [10,11].

## 2. Macrovascular Complications

The American Heart Association has designated T2D as one of the main risk factors for cardiovascular disease, which remains the leading cause of morbidity and mortality in patients with diabetes. Following a diabetes diagnosis, the risk of vascular disease increases by 73%, the risk of coronary artery disease by 100%, and the likelihood of ischemic cerebrovascular disease rises by 127% [10]. On average, the onset of these conditions in patients with T2D occurs 15 years earlier than in the general population [9].

Macrovascular disease, which affects large blood vessels, is characterized by a complex inflammatory process. When secondary to diabetes, its onset is marked by the development of chronic hyperglycemia and IR, both hallmarks of the disease [12]. Among the multiple pathophysiological processes involved are alterations in the metabolism of various substrates, the generation of glucotoxic and lipotoxic states, and increased oxidative stress. Collectively, these factors disrupt cardiovascular homeostasis, cause injury and dysfunction of the endothelium in both large and small vessels and arteries, and ultimately trigger the development of diverse cardiovascular complications [9,12].

Resistin, in addition to being a mediator of IR and an adipokine with pro-inflammatory properties, appears to be associated with an increased risk of cardiovascular diseases and their progression [13]. The international literature reports clinical studies in humans demonstrating an association between both systemic and local resistin expression and cardiovascular diseases. Furthermore, in vivo and in vitro studies have explored the cellular and molecular mechanisms through which resistin influences the progression of atherosclerosis and endothelial dysfunction. Broadly, resistin contributes to the perpetuation of dysglycemia characteristic of diabetes, promoting pro-inflammatory states and causing endothelial dysfunction. Hyperglycemia remains the primary determinant in the development of macrovascular and microvascular complications in T2D and their progression to severe outcomes [12].

From the clinical perspective, multiple clinical studies have investigated the role of resistin as a contributing factor in hypertension, atherosclerosis, coronary heart disease (CHD), acute myocardial infarction (AMI), and heart failure, and as an indicator of severe myocardial damage, poor prognosis, and a predictor of cardiovascular mortality in T2D. The findings of these studies will be discussed in detail.

### 2.1. Hypertension

Hypertension (HTN) affects approximately 70% of patients with T2D and it is a risk factor for the development of other cardiovascular complications such as coronary artery disease and cerebrovascular disease [12,14]. The relationship between T2D and HTN is closely intertwined. Pathophysiologically, T2D represents a state of chronic inflammation and oxidative stress that leads to endothelial dysfunction, vascular inflammation, increased arterial remodeling, and atherosclerosis. It is well known that T2D disrupts all the factors that regulate blood pressure, including cardiac output and systemic vascular resistance (SVR). Sodium retention, a hallmark of this metabolic disorder, promotes water retention, blood volume expansion, increased stroke volume, and consequently, elevated cardiac output. Studies have demonstrated that hyperglycemia in T2D leads to inappropriate activation of the renin–angiotensin–aldosterone system (RAAS), reduces nitric oxide (NO) availability, and promotes the formation of reactive oxygen species (ROS). These mechanisms contribute to endothelial dysfunction and the loss of vasodilation capacity. Additionally, arterial stiffness increases due to the accumulation of advanced glycation end products (AGEs), collagen, and extracellular matrix in the vascular walls. Furthermore, hyperglycemia elevates cytokine levels, including IL-1β, IL-6, and MCP-1, thereby exacerbating inflammation and increasing cardiovascular risk [15,16].

Accumulating evidence suggests that resistin influences blood pressure regulation; its levels are higher in patients with HTN compared to normotensive individuals, indicating that resistin may be a risk factor for the development of hypertension [14]. Although the cellular and molecular mechanisms through which resistin induces hypertension in diabetic individuals are not fully understood, some experimental studies in animal models have provided valuable insights.

In mice, it has been observed that resistin regulation of blood pressure appears to be dependent on Toll-like receptor 4 (TLR-4). Resistin acts by activating the renin–angiotensin system, leading to alterations in the production of various vasoconstrictors and vasodilators [15]. Another proposed mechanism is that resistin increases the expression and release of endothelin-1 (ET-1) and downregulates endothelial nitric oxide synthase in human cells, resulting in hypertension [16]. Additionally, resistin has been shown to induce endothelial damage by increasing oxidative stress through the activation of the p38/MAPK pathway, which promotes endothelial and vascular smooth muscle proliferation, disrupts the vascular monolayer, and increases vascular permeability [17].

In humans, TLR4 and resistin appear to play a significant role in the development of hypertension in T2D. A clinical study reported significantly elevated serum levels of TLR4 and resistin in hypertensive individuals with T2D compared to hypertensive patients without diabetes [18].

Hyper-resistinemia has been identified as a common feature in patients with hypertension [14]. In individuals with both T2D and hypertension, resistin levels are even higher and show a positive correlation with mean arterial pressure values [19]. Collectively, these findings suggest that resistin may represent a risk factor for the development of hypertension and could potentially be used as a biomarker for this condition.

Finally, a meta-analysis published in 2017 found that the correlation between serum resistin and hypertension differs across populations, being more consistent in Hispanic and Asian populations. This variability was attributed to differences in sample sizes and demographic characteristics among the studies included in the meta-analysis [14].

### 2.2. Atherosclerosis and Coronary Heart Disease (CHD)

Atherosclerosis is a fundamental pathological change underlying many cardiovascular diseases, characterized by the chronic and progressive accumulation of pro-inflammatory plaques composed of lipid and cellular deposits within arterial walls [13]. The endothelium of arterial blood vessels is constantly exposed to varying degrees of stress due to their irregular geometry and the physical forces generated by changes in blood flow throughout the arterial tree. This stress induces alterations in cellular morphology, signaling, and gene expression. In inflammatory states—such as obesity, insulin resistance, and type 2 diabetes—the presence of pro-inflammatory cytokines (including interleukins and TNF-α) disrupts the normal functioning of the glycocalyx and the production of nitric oxide (NO), prostacyclin, and endothelin. As a result, a vasoconstrictive and pro-coagulant environment is established. Additionally, the transport and molecular signaling in endothelial cells deteriorate; the expression of adhesion molecules such as E-selectin, ICAM, and VCAM increases; and the accumulation of leukocytes and modified lipoproteins (particularly LDL) in the subendothelial space is favored [20]. In addition, the hyperglycemia characteristic of T2D induces the overproduction of reactive oxygen species (ROS) in blood vessels, leading to DNA damage. In this environment, macrophages release enzymes that modify lipids, while ROS oxidize the lipoproteins accumulated in the intima. Endothelial dysfunction further increases arterial wall permeability, promoting the accumulation of lipoproteins, cholesterol, and inflammatory cells, ultimately triggering the atherosclerotic process [21,22].

A common feature shared by both atherosclerosis and T2D is inflammation. Pro-inflammatory adipokines have been identified as key contributors to the increased risk of atherosclerosis and may serve as useful biomarkers for its progression [23]. In particular, resistin appears to play a significant role in promoting vascular inflammation, lipid accumulation, and the destabilization of atherosclerotic plaques [12,13,23]. Experimental studies have demonstrated the pro-atherosclerotic effects of resistin on vascular smooth muscle and the endothelium [24]. Inducing resistin overexpression in the vascular tissues of rabbits with atherosclerosis leads to increased endothelial inflammation and the exacerbation of atherosclerotic plaques, driven by enhanced macrophage infiltration and monocyte adhesion to arterial walls [25].

Resistin also promotes elevated lipid profiles and IR, enhances macrophage polarization, and fosters inflammatory states through the expression of cytokines such as TNF-α and IL-6. At the endothelial level, resistin facilitates monocyte adhesion via the expression of endothelial adhesion molecules, including VCAM-1, VSMCs, and MCP-1. Additionally, resistin increases the mRNA and protein levels of the scavenger receptor class A (SR-A) and CD36 through the upregulation of AP-1 and PPARγ, two receptors involved in the uptake of modified LDL by macrophages; together, these mechanisms promote the formation of atheromatous plaques [26]. The previously cited findings support the hypothesis that resistin acts as an inflammatory mediator with a significant role in the development of atherosclerosis, as well as in the progression and instability of atherosclerotic plaques [23,27]. Previous studies have reported that resistin disrupts vascular system homeostasis by increasing the production of reactive oxygen species (ROS) and decreasing the availability of nitric oxide (NO) in coronary arteries across various animal models. Elevated levels of this adipokine have been detected in sclerotic lesions of coronary arteries [28,29].

Various clinical and epidemiological studies have explored the relationship between resistin and the onset of atherosclerosis in the context of T2D. In Korean subjects with T2D and CHD, serum resistin levels were significantly higher compared to those with T2D without a history of cardiovascular disease [30]. Additionally, elevated serum resistin was associated with an increased risk of CHD development in postmenopausal women with T2D, as its rise coincided with elevated inflammatory and endothelial dysfunction markers such as high-sensitivity C-reactive protein (hsCRP), total plasma homocysteine (tHcy), IL-6, and soluble vascular cell adhesion molecule (sVCAM) [26]. Furthermore, evidence suggests a positive association between resistin levels and inflammatory markers such as IL-6, soluble tumor necrosis factor receptor 2 (Sol TNF-R2), and lipoprotein-associated phospholipase A2 (Lp-PLA2), as well as increased coronary artery calcification in both healthy individuals and those with T2D [29].

In clinical settings, combining serum resistin measurement with high-frequency ultrasonography (HFUS) during carotid atherosclerosis (CA) assessment in individuals with T2D has been shown to improve the estimation of the vascular remodeling impact compared to the use of HFUS alone [31]. The relationship between resistin and another cardiovascular risk marker, carotid intima–media thickness (IMT), has also been studied. In a study conducted on 169 patients with and without T2D, IMT was measured and compared between the two groups. An elevation in serum resistin levels was found in the T2D group; however, no correlation was found between circulating levels of this adipokine and IMT [32].

The aforementioned experimental and clinical studies provide strong evidence supporting the role of resistin as a mediator in the progression of atherosclerosis and the development of CHD in patients with T2D.

### 2.3. Acute Myocardial Infarction and Myocardial Ischemia Reperfusion Injury

Ischemic heart disease is one of the leading causes of mortality worldwide. Type 2 diabetes (T2D) is a well-established risk factor for acute myocardial infarction (AMI), which is the primary cause of death in patients with this chronic metabolic disease [33]. AMI occurs due to an imbalance between oxygen supply and demand in the myocardium, and atherosclerosis plays a fundamental role in its pathogenesis. According to a meta-analysis, more than 70% of fatal heart attacks are caused by atherosclerotic plaque rupture [34].

As previously discussed, several factors contribute to the development of atherosclerosis, including chronic inflammation, oxidative stress, and endothelial dysfunction. However, in the context of AMI, hypercoagulability is a main pathological mechanism. Insulin resistance in T2D is associated with molecular pathways that establish a prothrombotic state, primarily through platelet and fibrin dysfunction and alterations in the expression of coagulation cascade factors, such as plasminogen activator inhibitor (PAI) and fibrinolysis inhibitor (FI) [35]. Platelet glucose uptake increases with hyperglycemia, while oxidative stress disrupts intracellular calcium homeostasis and thromboxane synthesis; coupled with an increased expression of procoagulant factors (tissue factor and factor VII) and decreased levels of anticoagulant agents (protein C and antithrombin), these mechanisms promote platelet aggregation and thrombus formation [36,37]. T2D leads to abnormal lipid metabolism through the oxidation and modification of LDL induced by hyperglycemia, which contributes to atheroma plaque formation and reduces its stability, further promoting vascular smooth muscle cell apoptosis [38].

Several in vitro and ex vivo studies conducted on human platelets and animal models have demonstrated that resistin promotes a prothrombotic state by enhancing platelet activation and aggregation through the p38 MAPK pathway and P-selectin expression [39,40]. Additionally, in vitro, resistin has been shown to increase factor Xa activity and elevate levels of key fibrinolytic mediators, such as plasminogen activator inhibitor-1 (PAI-1) and tissue plasminogen activator (tPA), in human umbilical vein endothelial cells (HUVECs) [36]. These findings may help clarify the mechanisms underlying the prothrombotic state observed in T2D.

Resistin has shown potential as a significant cardiovascular risk factor in both the general population and individuals with T2D. It has been established that the risk of acute myocardial infarction (AMI) is twice as high in individuals with elevated serum resistin levels compared to those with normal levels [41]. Additionally, hyper-resistinemia has been detected in patients with ischemic heart disease, independently of the presence of T2D [42]. Moreover, serum resistin levels were found to be elevated in patients with acute myocardial infarction (AMI) and identified as a risk factor for major adverse cardiovascular events (MACEs) in this group [43].

The association between serum resistin and several biochemical markers involved in AMI pathogenesis has been studied in patients with T2D. In a study conducted on patients with and without T2D who suffered a STEMI, serum resistin was negatively correlated with HDL cholesterol and positively correlated with troponin I and triglyceride levels; however, no association was found with serum creatine kinase (CK), lactate dehydrogenase (LDH), or the HOMA-IR index [44]. Additionally, elevated resistin levels have been positively correlated with increased serum tissue factor (TF) levels, a key component in thrombogenesis [36].

The therapeutic strategy for acute myocardial infarction (AMI) focuses on restoring blood flow as quickly as possible to limit myocardial damage and improve patient outcomes. However, the abrupt reintroduction of oxygen and nutrients to the ischemic tissue can also exacerbate cardiomyocyte damage and increase necrosis, a phenomenon known as myocardial ischemia/reperfusion injury (MIRI) [45]. The pathophysiology of MIRI involves oxidative stress, lipid and glucose metabolism dysregulation, inflammation, and calcium imbalance in myocardial cells; together, these mechanisms contribute to necrosis and apoptosis of cardiomyocytes. T2D therefore increases the heart’s vulnerability to this type of injury through several mechanisms; hyperglycemia promotes excessive ROS formation and insulin resistance disrupts myocardial metabolism and impairs angiogenesis and microvascular function, reducing the myocardium’s ability to withstand ischemia [46].

Regarding resistin, it has been suggested that it may have a cardioprotective effect by activating components of the RISK (Reperfusion Injury Salvage Kinase) signaling pathway, including PI3K, Akt, and PKC. This activation stimulates cell proliferation and reduces cardiomyocyte apoptosis [45,46]. However, the relationship between resistin and MIRI remains unclear, as findings from various experiments have been contradictory. While some studies indicate that resistin exerts a protective effect against ischemia and facilitates cardiac recovery after reperfusion, others have failed to replicate these results.

The cardioprotective effect of resistin was observed in an experiment using a mouse heart perfusion model, where pretreatment with resistin limited damage during ischemia by reducing cardiomyocyte apoptosis and the infarct size. Additionally, resistin facilitated functional recovery following reperfusion by activating the RISK signaling pathway involving PI3K/Akt/PKC [47]. Conversely, preconditioning with recombinant human resistin prior to ischemia induction resulted in deleterious effects on contractile function during the reperfusion phase, along with a significant increase in ANP, BNP, CK, and TNF-α levels. In the same experiment, the co-administration of resistin with a TNF-α inhibitor improved myocardial recovery and reduced the expression of the other biochemical markers [48]. Furthermore, the administration of resistin during the reperfusion phase in another study using a similar I/R injury model in mice and in vitro human atrial tissue showed no effect on the infarct size in either model. This was observed despite Western blot results in human atrial cells demonstrating increased phosphorylation of Akt at serine and threonine residues in response to exogenous resistin administration [49]. From these findings, it can be concluded that although resistin has been shown to induce Akt phosphorylation, this effect does not translate into cardioprotection. On the contrary, hyper-resistinemia not only fails to limit ischemic damage to the myocardium but also appears to impair post-ischemic recovery through its influence on TNF-α signaling pathways. This mechanism adds to the already well-established deleterious effects of resistin on insulin signaling and lipid metabolism, which are characteristic of type 2 diabetes (T2D).

There is evidence supporting the involvement of resistin in the pathophysiology of myocardial ischemia and reperfusion injury in T2D; however, its exact cellular and molecular mechanisms remain controversial. Further research is essential to clarify resistin impact on the onset of myocardial injury and its potential utility as a biochemical marker in clinical practice.

### 2.4. Heart Failure

Heart failure (HF) is a structural and/or functional impairment of the heart that manifests as a clinical syndrome, along with elevated biomarkers such as brain natriuretic peptide (BNP) and objective evidence of congestion, which may affect only the lungs or the entire circulatory system [50]. T2D significantly increases the risk of heart failure and other cardiovascular disorders, which are the leading causes of death in diabetic patients. The risk of developing HF in individuals with T2D is twice as high in men and up to five times higher in women compared to non-diabetic individuals. Moreover, this risk increases in direct proportion to the severity of T2D [51].

Type 2 diabetes (T2D) induces structural and functional alterations in the heart through complex pathophysiological mechanisms that are not yet fully understood. Among the main identified mechanisms are glucotoxicity and lipotoxicity, which affect both the vascular walls of the heart and the cardiac muscle itself. One key process is the formation of advanced glycation end-products (AGEs), which result from the non-enzymatic glycation of proteins and lipids under conditions of sustained hyperglycemia or other pathological states such as hypoxia, ischemia, or reperfusion. These AGEs interact with their specific receptor (RAGE), triggering an inflammatory and oxidative stress response by upregulating cytokine expression, adhesion molecules (VCAM and ICAM), and ROS. This cascade leads to monocyte infiltration into the arterial walls and impaired vasodilation due to reduced NO and ET-1 levels, ultimately resulting in endothelial dysfunction. Additionally, the oxidation and glycation of low-density lipoproteins (LDLs) increase their density and promote their accumulation in the arterial intima–media layer. These modified LDL molecules are subsequently phagocytized by macrophages, forming foam cells and releasing pro-inflammatory cytokines such as TNF-α, IL-1β, and IL-6. This inflammatory environment, along with epigenetic modifications in gene expression and changes in the regulation of microRNAs and long non-coding RNAs, contributes to the progression of heart failure [52,53].

Resistin has been implicated in increased oxidative stress and systemic inflammation and is believed to induce chronic myocardial damage through the formation of ROS, pro-inflammatory cytokines, and mitochondrial dysfunction in cardiomyocytes. As a result, cardiac muscle cell dysfunction leads to necrosis, atrial remodeling, loss of myocardial contractility, left ventricular remodeling, and ultimately, heart failure [54]. Resistin also influences cardiac hypertrophy and dysfunction by interfering with glucose transport through the disruption of endosomal vesicle trafficking [55]. The disruption of vascular homeostasis produced by resistin occurs through an increased ROS production and decreased NO availability in coronary arteries, as observed in various animal models where elevated resistin expression has been detected in sclerotic coronary artery lesions [28,29]. Experimental results from cardiomyocytes isolated from diabetic rat hearts suggest that Ca^2+^ dyshomeostasis and altered Ca^2+^/ATPase (Serca2) activity are key mechanisms driving the upregulation of resistin expression in this metabolic disorder [56]. Additionally, resistin activates cell signaling pathways such as STAT3, Akt, ERK1/2, and MAPK in cardiomyocytes, reducing contractility and altering the speed of the contraction and relaxation phases of the cardiac cycle, thereby contributing to cardiac hypertrophy [57]. These findings suggest a direct link between cardiac dysfunction in T2D and resistin levels

Clinically, there are few published studies exploring the relationship between serum resistin and heart failure in patients with T2D. It has been shown that this adipokine is elevated in patients with evidence of systolic dysfunction compared to healthy individuals, and this increase is associated with the left ventricular mass index (LVMI) and other echocardiographic parameters used to assess cardiac function [58]. Additionally, elevated serum resistin levels have been associated with worse functional status in heart failure patients according to the New York Heart Association (NYHA) classification [59].

Due to the limited clinical evidence, the utility of measuring serum resistin levels as an indicator of heart failure progression and prognosis lacks sufficient support to justify its use in routine clinical practice. Therefore, further studies with larger sample sizes and diverse populations are needed to provide more robust evidence on this topic.

## 3. Microvascular Complications

The presence of microvascular complications in T2D not only increases mortality but also significantly deteriorates the quality of life of patients through the development of nephropathy and retinopathy [9].

Several factors are implicated in the development of diabetic microangiopathy, including hyperglycemia-induced activation of the polyol pathway, increased protein kinase C activity, and the excessive production of ROS and advanced glycation end products (AGEs) [8]. Resistin also appears to be involved in the pathophysiology of diabetes-related microvascular complications. Serum resistin levels have been associated with the severity of diabetic retinopathy, nephropathy, and neuropathy. Furthermore, a positive correlation has been observed between serum resistin levels and both the number and severity of microangiopathies, independent of other factors such as sex, age, body mass index (BMI), and the duration of T2D [60].

### 3.1. Diabetic Nephropathy

T2D is the most significant individual risk factor for the development of end-stage renal disease (ESRD) in developed countries. Diabetic nephropathy (DN) presents as chronic kidney disease (CKD), characterized by a reduced glomerular filtration rate (GFR) and elevated urinary albumin excretion due to renal damage induced by poor long-term glycemic control [61].

The pathogenesis of DN is complex, multifactorial, and not yet fully understood. However, it is known to result from various disruptions in homeostasis, including hemodynamic changes, metabolic disorders, abnormalities in the synthesis of hormones such as angiotensin II (Ang-II), and alterations in metabolic or cellular signaling pathways. Although the exact mechanisms underlying DN have not been fully elucidated, several key factors have been identified. These include the involvement of the RAAS, the formation of AGEs, and the activation of molecular pathways such as transforming growth factor-beta 1 (TGF-β1), connective tissue growth factor (CTGF), protein kinase C (PKC), mitogen-activated protein kinases (MAPKs), and ROS. These pathways contribute to renal damage through various mediators and interact with each other in a complex network of pathological processes [62,63].

The pathophysiological mechanisms through which resistin contributes to the development of DN have not been fully elucidated. However, it is proposed that the elevated resistin levels associated with T2D lead to glomerular and renal dysfunction through adipokine’s pro-inflammatory effects, oxidative stress, and chronic inflammation [61,64]. In molecular studies, resistin mRNA expression has been quantified in patients with T2D and DN, showing significantly higher levels compared to healthy individuals [65]. Serum resistin levels have also been analyzed in conjunction with renal function in patients with DN, revealing significantly elevated resistin mRNA expression and serum levels in individuals with T2D and DN compared to both healthy subjects and patients with T2D without DN [61,65].

Multiple epidemiologic studies have demonstrated a negative correlation between resistin levels and the GFR in patients with T2D [61,66,67,68]. For every standard deviation (SD) increase in serum resistin levels, the risk of a reduced GFR increases by 22% [67]. Resistin has been suggested as an independent predictive factor for the progression of renal disease in DN. Patients with T2D and higher resistin levels are up to eight times more likely to develop DN, and the highest resistin levels are observed in individuals with more advanced stages of DN [67,69]. Additionally, resistin has been shown to predict renal function decline and hospital admissions in diabetic patients with CKD [70].

Accumulating evidence suggests that elevated resistin levels are directly associated with increased microalbuminuria and serum creatinine in patients with a reduced GFR. Moreover, patients undergoing peritoneal dialysis and hemodialysis exhibit significantly higher serum resistin levels compared to those receiving conservative treatment [71,72].

The evidence from these studies supports the notion of a direct correlation between DN and resistin, suggesting that elevated resistin levels may be a significant risk factor for the development and progression of DN in T2D. Therefore, quantifying serum resistin levels could be useful as a biomarker for assessing this T2D-related complication.

### 3.2. Diabetic Retinopathy

Diabetic retinopathy (DR) is the most common complication of T2D, characterized by progressive damage to the retinal microvasculature, which can ultimately lead to blindness. This damage is secondary to inflammatory processes, ischemia, and retinal neovascularization [73].

In T2D, prolonged hyperglycemia leads to oxidative stress, inflammation, and activation of the renin–angiotensin system, which impairs endothelial function and causes pericyte degeneration. This weakens the blood–retinal barrier (BRB), increasing vascular permeability in the retina. Additionally, the accumulation of AGEs and the overexpression of vascular endothelial growth factor (VEGF-A) stimulate the formation of abnormal new blood vessels, contributing to the development of diabetic macular edema (DME). The activation of microglia and Müller glial cells further intensifies the inflammatory response, leading to increased damage to retinal neurons and blood vessels, ultimately resulting in vision loss in affected patients [74].

Experimental studies have demonstrated that resistin promotes angiogenesis in endothelial cells through the upregulation of vascular endothelial growth factor receptor 2 (VEGFR2), suggesting that elevated levels of this adipokine may contribute to the development of DR, particularly the proliferative form [75].

There are few studies in the international literature investigating the relationship between DR and resistin levels. The exact cellular and molecular mechanisms involved remain unclear, as does the clinical relevance of resistin quantification. The available clinical evidence is contradictory; some authors have reported no significant differences in serum resistin levels between patients with T2D and those with T2D and proliferative DR [71]. In contrast, other studies have shown a positive correlation between hyper-resistinemia and DR severity, with the highest resistin levels observed in patients with more advanced stages of proliferative DR [61,72].

Given the limited number of experimental and clinical studies in humans, it is premature to conclude that resistin can serve as a biomarker or prognostic factor for DR. Additionally, the lack of knowledge regarding the underlying cellular and molecular mechanisms makes it unclear as to whether this adipokine plays a significant role in the pathogenesis of this microvascular complication of T2D. This knowledge gap presents a valuable opportunity for future clinical and preclinical research.

The details of the design, sample size, and results of the previously cited studies are summarized in Table S1.

## 4. Conclusions

Since its discovery in 2001, resistin has gained significant interest within the scientific community due to its potential role in the development of IR and T2D. In recent years, the pathophysiological mechanisms by which resistin contributes to IR and T2D have been extensively studied in murine models, both in vitro and in vivo. However, resistin has been less explored in clinical settings.

Regarding the pathophysiology of macrovascular and microvascular complications of T2D in humans, although alterations in resistin levels have been observed in affected individuals, the exact molecular mechanisms remain unclear. Various clinical studies attempting to correlate resistin with the progression and severity of chronic T2D complications have reported a tendency toward an increased risk of these conditions in individuals with elevated resistin levels compared to healthy subjects. Nevertheless, the current evidence is insufficient to support the use of resistin as a biomarker in clinical practice. Further clinical and epidemiological studies across diverse populations are needed to determine whether a definitive correlation exists between serum resistin levels and the onset or progression of chronic T2D complications.

This comprehensive review analyzed published experimental and clinical studies on the role of resistin in major macrovascular and microvascular diseases, specifically within T2D. Unlike previous reviews that have examined and discussed resistin’s role in isolated diseases, this study focuses exclusively on diabetes-induced models and clinical evidence in patients with T2D, providing a more targeted perspective on its pathophysiological mechanisms and potential as a clinical biomarker. However, this review faces several limitations due to the heterogeneity of the included studies, which present differences in models, populations, and methodologies, making direct comparisons of results challenging. Variability in resistin measurement techniques affects reproducibility and limits its validation as a biomarker. Although experimental evidence supports resistin’s role in the pathogenesis of diabetic complications, clinical studies have yet to confirm its diagnostic or prognostic utility. Additionally, the lack of well-designed clinical trials and the exclusive focus on studies published in English may have led to the exclusion of relevant information. Finally, this review specifically compiled evidence related to vascular complications; however, other conditions closely associated with T2D—such as dyslipidemia, Alzheimer’s disease, and diabetic neuropathy—were excluded from this review despite resistin’s potential involvement in their pathophysiology. These topics could be explored in future reviews.

In conclusion, resistin appears to play an important role in the development and progression of chronic T2D complications in both humans and animal models. However, the precise mechanisms involved have not yet been fully elucidated. In clinical practice, there is currently insufficient evidence to justify the use of resistin as a biochemical marker for the diagnosis or prognosis of chronic T2D complications.

## Supplementary Materials

The following supporting information can be downloaded at: https://www.mdpi.com/article/10.3390/life15040585/s1, Table S1: Clinical and Epidemiological Studies Providing Evidence on the Role of Resistin in Macrovascular and Microvascular Complications of Type 2 Diabetes (T2D). References [76,77,78,79] are cited in the Supplementary Materials.

## Abbreviations

The following abbreviations are used in this manuscript:

T2D	Type 2 Diabetes
IR	Insulin Resistance
CHD	Coronary Heart Disease
HTN	Hypertension
NO	Nitric Oxide
ROS	Reactive Oxygen Species
ET-1	Endothelin-1
MAPK	Mitogen-Activated Protein Kinase
VCAM-1	Vascular Cell Adhesion Molecule-1
VSMCs	Vascular Smooth Muscle Cells
MCP-1	Monocyte Chemoattractant Protein-1
HFUS	High-Frequency Ultrasonography
HF	Heart Failure
LVMI	Left Ventricular Mass Index
NYHA	New York Heart Association
ESRD	End-Stage Renal Disease
DN	Diabetic Nephropathy
CKD	Chronic Kidney Disease
GFR	Glomerular Filtration Rate
BMI	Body Mass Index
SD	Standard Deviation
DR	Diabetic Retinopathy
VEGFR2	Vascular Endothelial Growth Factor Receptor 2
AGEs	Advanced Glycation End Products
hsCRP	High-Sensitivity C-Reactive Protein
tHcy	Total Plasma Homocysteine
Sol TNF-R2	Soluble Tumor Necrosis Factor Receptor 2
Lp-PLA2	Lipoprotein-Associated Phospholipase A2
SR-A	Scavenger Receptor Class A
PAI-1	Plasminogen Activator Inhibitor 1
tPA	Tissue Plasminogen Activator
CK	Creatine Kinase
LDH	Lactate Dehydrogenase
PKC	Protein Kinase C
RISK	Reperfusion Injury Salvage Kinase
PI3K	Phosphoinositide 3-kinase
HUVECs	Human Umbilical Vein Endothelial Cells
STEMI	Acute ST Segment Elevation Myocardial Infarction
MACE	Major Adverse Cardiovascular Events
MIRI	Myocardial Ischemia/Reperfusion Injury
