# Supplementary material for: The Role of Resistin in Macrovascular and Microvascular Complications of Type 2 Diabetes
Source: Life (Basel). 2025 Apr 2;15(4):585. doi: 10.3390/life15040585 (PMC12028410; doi:10.3390/life15040585)
Supplement: Supplementary file 1 [file life-15-00585-s001.zip › life-3490654-supplementary.pdf]

TABLES

Table S1: Clinical and Epidemiological Studies Providing Evidence on the Role of Resistin in Macrovascular and Microvascular Complications of Type 2 Diabetes (T2D)

Type of complication	Authors and Year	Study Design	Variables Measured	n	Population	Results
Macrovascular Complications						
Hypertension	Takata et al 2008 [19]	Analytical Cross-Sectional	Serum resistin, systolic BP, diastolic BP, CIMT	2010	Japanese (Unspecified ethnicity)	Positive correlation between serum resistin and systolic BP (r=0.29, p<0.01), diastolic BP (r=0.21, p<0.05), and CIMT (r=0.27, p<0.05). Resistin identified as an independent risk factor for hypertension in T2DM patients (p<0.05).
	Rubio et al., 2013 [76]	Analytic Cross-Sectional	Serum resistin, adiponectin	30	Mexican (Unspecified ethnicity)	Significantly higher resistin levels in subjects with hypertension and T2DM vs controls (p<0.01). Positive correlation between CIMT and resistin (R=0.45, p<0.02). Positive correlation between CIMT and ARI (R=0.58, p<0.001).
	Rubio et al., 2014 [77]	Analytic Cross-Sectional	Serum resistin	80	Mexican (Unspecified ethnicity)	Presence of hypertension and T2DM significantly increases resistin levels compared to T2DM patients without hypertension (p<0.005).

	Zhang et al., 2017 [14]	Systematic Review and Meta-analysis	Serum resistin	1363	Multiple populations (European: 57.4%, Asian: 38.3%, Hispanic: 4.2%)	Statistically significant association between serum resistin and T2DM with hypertension vs healthy controls (SMD=1.20, 95% CI: 0.32-2.07, p=0.008).
	Al-Taie et al., 2021 [18]	Analytic Cross-Sectional	Serum resistin, TLR4	120	Iraqi (Unspecified ethnicity)	Higher levels of resistin and TLR4 in both organ groups compared to healthy controls (p<0.001).
Atherosclerosis and CHD	Reilly et al., 2005 [29]	Analytic Cross-Sectional	Serum resistin, TNF-R2, IL-6, Lp-PLA2, CAC	215	American (Caucasian: 95%)	Positive association between plasma resistin and TNF-R2 (p<0.001), IL-6 (p=0.04), and Lp-PLA2 (p=0.02). Increased CAC in healthy subjects and those with T2DM (OR 1.23, p=0.03).
	On et al., 2007 [30]	Analytic Cross-Sectional	Serum resistin	92	Korean (Unspecified ethnicity)	Higher resistin levels in subjects with DMCAD compared to T2DM without CHD (p<0.001).
	Dullaart et al., (2007) [32]	Case-Control	Serum resistin, adiponectin and leptin, hsCRP, TNF-α Carotid IMT	169	Dutch (Caucasian)	Higher resistin in diabetic patients vs controls (p= 0.003). Carotid IMT was not correlated with serum resistin.
	Giandalia et al., 2021 [78]	Analytic Cross-Sectional	Serum resistin, hsCRP, IL-6, sVCAM	187	Italian (Unspecified ethnicity)	Higher serum resistin, IL-6, and sVCAM levels in women with CHD and T2DM compared to T2DM without CHD (p<0.001).

Acute myocardial infarction (AMI)	Bobbert et al. 2011 [36]	Case-Control	Serum leptin and resistin. Plasma TF	62	German (Unspecified ethnicity)	Increased resistin in T2D patient's vs controls ($p<0.05$). Positive correlation between resistin and plasma TF ($r = 0.373$, $p < 0.05$)
	Korah et al. 2011 [44]	Case-Control	Serum resistin, troponin I, CK, LDH and HOMA-IR index.	55	Egyptian (Unspecified ethnicity)	Resistin correlated with troponin I ($r=0.59$, $p<0.05$), triglycerides ($r=0.47$, $p<0.05$) and negatively with HDL ($r=-0.46$, $p<0.05$) in patients with T2D and STEMI. No association with CK, LDH and HOMA-IR index.
	Yaseen et al. 2012 [42]	Case-Control	Serum resistin and IL-6	147	Pakistan (Unspecified ethnicity)	Increased resistin and IL-6 in diabetics and non-diabetics with ischemic heart disease. Resistin correlation with ischemic heart disease ($r=0.859$, $p=0.001$).
Heart Failure	Takeshi et al., 2007 [59]	Prospective Cohort	Serum resistin, cardiac event rate	126	Japanese (Unspecified ethnicity)	Elevated resistin is a predictor of cardiac events in subjects with CHF (HR: 1.439, 95% CI: 1.017-2.059, $p=0.0414$).
	Ghanem et al., 2020 [58]	Case-Control	Serum resistin, IL-6, NT-proBNP, LVMI	150	Egyptian (Unspecified ethnicity)	Higher LVMI in T2DM group compared to healthy controls ($p<0.001$). Positive association of LVMI with NT-proBNP ($r=0.570$, $p<0.001$), resistin ($r=0.495$, $p<0.05$), and IL-6 ($r=0.496$, $p<0.005$).

Microvascular Complications						
Diabetic Nephropathy	Osawa et al., 2007 [60]	Analytic Cross-Sectional	Serum resistin, microangiopathy severity (retinopathy, nephropathy, neuropathy)	238	Japanese (Unspecified ethnicity)	Positive correlation between serum resistin levels and severity of retinopathy (p=0.011), nephropathy (p=0.011), and neuropathy (p=0.005).
	Moreno et al., 2015 [67]	Analytic Cross-Sectional	Serum resistin, eGFR	1560	Italian-Americans (Unspecified ethnicity)	Inverse association between resistin increase (1 SD) and eGFR (p<0.001).
	Bonito et al., 2019 [70]	Prospective	Serum resistin	78	Portuguese (Unspecified ethnicity)	Positive correlation between resistin levels and hospital admissions for CVD in subjects with T2DM and CKD (OR=2.074, 95% CI: 1.55-7.29, p=0.047).
	Tofancshihua et al., 2020 [68]	Case-Control	Serum resistin, GFR	89	Chinese (Unspecified ethnicity)	Higher resistin levels in subjects with T2DM and DN compared to T2DM and healthy controls (p<0.05). Slight inverse correlation between GFR and resistin (r=-0.233, p=0.031).
	Li et al., 2021 [61]	Analytic Cross-Sectional	Serum resistin, ANGPTL-8	278	Chinese (Unspecified ethnicity)	Positive correlation between resistin levels and T2DM with DN (r=0.186, p<0.05). Resistin identified as a risk factor for DN (OR=2.4, 95% CI: 1.48-4.20, p=0.001).

	Huang et al., 2021 [69]	Analytic Cross-Sectional	Serum resistin, HOMA-IR	309	Chinese (Unspecified ethnicity)	Higher serum resistin levels in subjects with T2DM as a risk factor for DN development (OR=8.29, p<0.001).
Diabetic Retinopathy	Azab et al., 2016 [79]	Analytic Cross-Sectional	Serum resistin, DR stage	50	Egyptian (Unspecified ethnicity)	No significant correlation between serum resistin and DR stage (p=0.319).
	Gurlevik et al., 2019 [75]	Analytic Cross-Sectional	Serum resistin, vitreous humor resistin	67	Turkish (Unspecified ethnicity)	No statistical significance between serum resistin levels in subjects with T2DM vs T2DM with DR (p>0.05). No detectable resistin levels in vitreous humor.

Abbreviations:

ARI: adiponectin/resistin index, SMD: standard mean difference, DMCAD: diabetes with coronary artery disease, CHD: coronary heart disease, tHcy: total homocysteine, sVCAM: soluble vascular cell adhesion molecule, hsCRP: high-sensitivity C-reactive protein, CAC: coronary artery calcification, CIMT: carotid intima-media thickness, NT-proBNP: N-terminal pro b-type natriuretic peptide, LVMI: left ventricular mass index, USG: ultrasonography, ANGPTL-8: angiopoietin-like protein 8, DN: diabetic nephropathy, GFR: glomerular filtration rate, CVD: cardiovascular disease, CKD: chronic kidney disease, DR: diabetic retinopathy, HTN: hypertension, CHF: congestive heart failure, IMT: intima-media thickness.
